# Diagnostic accuracy of contrast-enhanced dynamic CT for small hypervascular hepatocellular carcinoma and assessment of dynamic enhancement patterns: Results of two-year follow-up using cone-beam CT hepatic arteriography

**DOI:** 10.1371/journal.pone.0203940

**Published:** 2018-09-19

**Authors:** Ye Ra Choi, Jin Wook Chung, Mi Hye Yu, Myungsu Lee, Jung Hoon Kim

**Affiliations:** 1 Department of Radiology, Seoul National University-Seoul Metropolitan Government Boramae Medical Center, Seoul, Korea; 2 Department of Radiology, and Institute of Radiation Medicine, Seoul National University College of Medicine, Seoul, Korea; 3 Department of Radiology, Konkuk University School of Medicine, Seoul, Korea; Ospedale San Raffaele, ITALY

## Abstract

**Objective:**

To evaluate the accuracy of CT for small, hypervascular hepatocellular carcinomas (HCCs) and assess the enhancement patterns on CT.

**Materials and methods:**

Ninety-nine patients who underwent cone-beam CT hepatic arteriography (CBCT-HA) during initial chemoembolization for HCC suspected on CT were enrolled in this study. A total of 297 hypervascular HCCs (142 ≥ 1 cm, 155 < 1 cm) were confirmed as HCCs based on two-year follow-up CT and CBCT-HA images. During the two-year follow-up, pre-existing hypervascular foci on CBCT-HA were regarded as HCCs at the initial presentation. Two radiologists categorized HCCs according to the following enhancement patterns on CT: type I, arterial enhancement and washout; type II, arterial enhancement without washout; and type III, no arterial enhancement. Two blinded reviewers rated the possibility of HCC.

**Results:**

For the 297 HCCs, the enhancement patterns according to size were as follows: type I ≥1 cm in 114 HCCs; type I <1 cm in 40 HCCs; type II ≥1 cm in 16 HCCs; type II <1 cm in 37 HCCs; type III ≥1 cm in 12 HCCs; and type III <1 cm in 10 HCCs. The remaining 68 HCCs (22.9%) were not detected on CT. The detection rates of HCCs ≥ 1 cm were 83.1%, 76.8%, and 83.1% in the formal report for reviewer 1 and reviewer 2. In comparison, the detection rates of HCCs < 1 cm were 20.6%, 17.4%, and 17.4% in the formal report for reviewer 1 and reviewer 2.

**Conclusion:**

Man**y** subcentimeter sized hypervascular HCCs were frequently missed or not evident on CT at the initial diagnostic workup. CT has limitations for diagnosing HCCs that are <1 cm in size or have atypical enhancement patterns.

## Introduction

With an enhanced understanding of the pathophysiology of hepatocellular carcinoma (HCC) and advances in imaging techniques, the diagnosis of HCC is increasing based on imaging criteria. Contrast-enhanced, dynamic computed tomography (CT) is one of the most important imaging techniques for diagnosing HCC in patients with cirrhosis, and this importance is attributable to accessibility and the well-established protocol standardization in many countries. According to the recent guidelines, including those of the American Association for the Study of Liver Diseases (AASLD) [1] and the European Association for the Study of the Liver (EASL) [2], contrast-enhanced, dynamic CT is considered the standard diagnostic technique for HCC in addition to magnetic resonance imaging (MRI). Although recent advances in the use of tissue-specific MRI contrast agents have been determined to be more sensitive for the detection of HCCs less than 2 cm in size [3, 4], the use of MRI rather than CT is limited by its relatively high cost and technical demand.

Studies have evaluated the performance of contrast-enhanced, dynamic CT for diagnosing HCC as a standard of reference rather than using histological examinations [5–12]. Contrast-enhanced, dynamic CT is a useful imaging modality for the diagnosis of HCCs larger than 1 cm, and the reported sensitivity is as high as 94% [7]; however, previous studies have reported various ranges with low sensitivity values for contrast-enhanced, dynamic CT for diagnosing HCCs less than 1 cm. The sensitivities for diagnosing small HCCs were particularly low due to difficulty in the detection and characterization of small nodules with atypical enhancement. In addition, pathologic examination either by biopsy or surgical resection as a reference standard has limitations due to technical difficulty. Even pathologic correlation in whole-liver explants is limited for detecting small HCCs less than 1 cm in diameter.

The early diagnosis of HCC remains a key goal for improving patient prognosis. Previous studies have shown that the smaller the HCC, the less likely there is to be microscopic vascular invasion [13, 14] and the more likely it is for local ablation to be complete [15, 16]; therefore, the early diagnosis of HCC is important for improving the prognosis. With the development of cone-beam CT hepatic arteriography (CBCT-HA) technology using a flat-panel detector, the detection of hypervascular HCC nodules has increased when used in conjunction with standard digital subtraction angiography (DSA) during transcatheter arterial chemoembolization (TACE)[17, 18]. Because CBCT-HA provides high lesion-to-background contrast and spatial resolution, it can be better than CT for the visualization of tumor-feeding vessels and provide information regarding tumor vascularity [19–23].

In this study, we assessed the diagnostic accuracy of contrast-enhanced, dynamic CT for small, hypervascular HCCs confirmed by the findings based on a two-year follow-up CT and CBCT-HA images, and assessed the dynamic enhancement patterns.

## Materials and methods

### Patient selection

**Fig 1:**
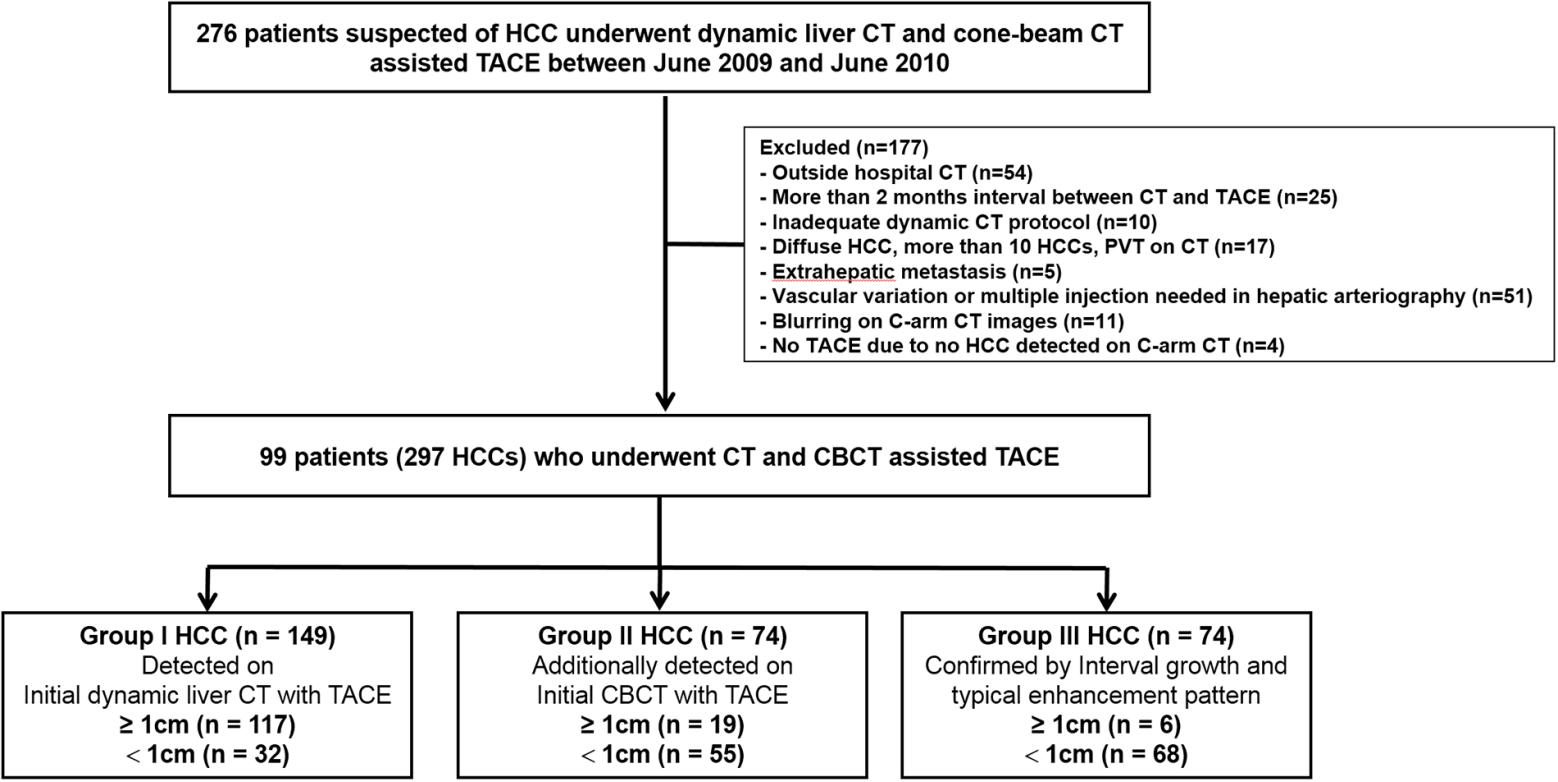
Flowchart shows study group inclusion process.

This retrospective study was approved by our institutional review board in Seoul National University Hospital (IRB No. 1707-044-868), and informed consent was waived. From June 2009 to June 2010, 276 patients suspected of having HCC underwent CT and CBCT-HA-assisted TACE. The final study group consisted of 99 patients (81 men and 18 women, mean age 63.6 ± 8.3) with 297 HCCs after excluding 177 patients who underwent CT at an outside hospital, had CT with more than a two-month interval between TACE, had an inadequate image quality, had more than 10 HCC lesions, and exhibited portal vein thrombosis or extrahepatic metastasis (Fig 1). The mean time interval between the initial CT and the CBCT-HA was 19 days (0–59 days).

### Diagnosis of HCC

To confirm the diagnosis hypervascular HCCs, two radiologists with four and 20 years of clinical experience in interventional radiology carefully reviewed the initial CT and CBCT-HA images and the follow-up images during the two years. We categorized the HCCs into three groups based on the detection of HCC. Group 1 (117 HCCs ≥ 1 cm and 32 HCCs < 1 cm) was categorized according to hypervascular HCC diagnosed on the initial CT and was divided into two subgroups. First, the lesions ≥ 1 cm and with the typical enhancement patterns of HCC such as arterial enhancement and portal/delayed washout seen on dynamic CT images, were diagnosed as HCC according to the AASLD guidelines [1]. Second, for the lesions < 1 cm or that had atypical enhancement patterns, two additional criteria were applied: (a) hyperenhancement seen on CBCT-HA; and (b) dense, compact iodized oil uptake (Lipiodol; Andre Guerbet, Aulnay- Sous Bois, France) was seen on the follow-up CT. Group 2 (19 HCCs ≥ 1 cm, 55 HCCs < 1 cm) exhibited hypervascular HCC diagnosed on the initial CBCT-HA and follow-up CT. In this patient group, the nodules had not been detected or suspected as being HCC on the initial CT but showed hyperenhancement on CBCT-HA and dense, compact iodized oil uptake on the follow-up CT. Group 3 (6 HCCs ≥ 1 cm, 68 HCCs < 1 cm) referred to hypervascular HCC diagnosed on the follow-up CBCT-HA and CT or MRI. This group included hypervascular nodules seen on the initial CBCT-HA but were observed to have been enlarged at the time of the follow-up CBCT-HA. At that time, the hypervascular nodules showed the typical enhancement patterns of HCC on both CT and MRI.

### CT techniques

In all 99 patients, triphasic or quadruple-phase (precontrast, arterial, portal venous, and delayed phase), contrast-enhanced, dynamic CT was performed at our hospital. All CT scans were obtained using one of the following MDCT scanners: 64-channel MDCT (Brilliance 64; Philips Medical Systems, Cleveland, OH, USA, n = 37); 16-channel (Sensation 16; Siemens Medical Systems, Forchheim, Germany, n = 15); 8-channel (LightSpeed Ultra; GE Healthcare, Milwaukee, WI, USA, n = 25); 4-channel (Mx8000; Philips Medical Systems, Cleveland, OH, USA. n = 4); 128-channel (Somatom Definition; Siemens Medical Systems, Forchheim, Germany [n = 17] or iCT; Philips Medical Systems, Cleveland, OH, USA [n = 1]). The scanning parameters were as follows: detector configuration, 8x1.25, 16x1.5, and 64x0.625 mm; slice thickness, 2.5, 3.0 and 3.0 mm; reconstruction interval, 2.5, 3.0, and 3.0 mm; table speed, 13.5, 24.0, and 46.9 mm per rotation; 250, 200, and 175 mA effective current; rotation time, 0.5, 0.5, and 0.75 s; tube potential 120 kVp; and matrix size, 512x512. CT images were obtained after an injection of iopromide (Ultravist 370; Bayer-Schering Pharma AG, Berlin, Germany) with a dose of 1.5 mL/kg (555 mg I/kg) per body weight followed by an injection of 30–40 mL of normal saline solution at a rate of 3.0–4.0 ml/s using an automatic power injector (Envision CT injector; Medrad, Pittsburgh, PA, USA). Arterial phase imaging was performed 15–19 s after achieving 100 HU attenuation of the descending aorta measured using a bolus tracking method. A 30 to 33 s delay after the arterial phase was obtained for portal venous phase acquisition. The delay time was 180 s for the equilibrium phase imaging following the administration of a contrast medium.

### CBCT-HA acquisition during TACE

All TACE procedures were performed using a CBCT-HA-capable angiography unit (AXIOM Artis dTA/VB30, Siemens, Erlangen, Germany). CBCT-HA images of the hepatic artery were obtained during a single breath-hold, and with 211° of circular trajectory for eight seconds. Contrast-enhanced images were acquired using undiluted, iodinated contrast medium (Pamiray 300, Dongkook Pharmaceutical, Seoul, Republic of Korea) at a flow rate of 1.5–6 ml/s for 12 seconds with a 4- or 6-second X-ray delay. The CBCT-HA acquisition protocol was as follows: 0.5° increment; 512 x 512 matrix in projections; 210° total angle at approximately 26° per second; a system dose of approximately 0.36 μGy per frame; and a total of 419 projections. The images obtained were immediately transferred to a dedicated workstation (Leonardo with Dyna CT; Siemens Healthcare) that promptly reconstructed them with a section thickness of 0.4 mm. If there was anatomic variation in the hepatic artery, such as the left hepatic artery coming from the left gastric artery and the right hepatic artery arising from the superior mesenteric artery, three-dimensional, rotational, cone-beam angiographic images of the left and right hepatic arteries were obtained separately. As a result, the entire liver was fully covered in the scanning range for all of the patients.

### Image analysis

Two abdominal radiologists with 15 and six years of clinical experience independently interpreted each patient’s initial CT images. The reviewers were informed that the patients could have HCC lesions although they were blinded to the number and location of the lesions. The reviewers independently recorded the size and location of the hepatic lesions they discovered. For multiple lesions in each patient, the reviewers indicated the image number and provided comments regarding each lesion on the review sheet in order to avoid confusion during data analysis. Based on the CT findings, the reviewers graded the possibility of HCC using a 5-point confidence scale. Grade 1 referred to definitely benign, wedge-shaped lesions with the base along the liver surface, a small portal branch within the lesion, hypervascular lesion in the arterial phase, and isoattenuation in the portal-delayed phases. Grade 2 referred to probably benign lesions that showed triangular areas or appeared as irregularly shaped, subtle, hypervascular lesions in the arterial phase and with isoattenuation in the portal-delayed phases. Grade 3 referred to indeterminate lesions. Grade 4 referred to probable HCC that appeared as an irregular, ill-defined, weak, hypervascular lesion in the arterial phase and with subtle hypoattenuation in the portal-delayed phases. Grade 5 referred to definite HCCs that appeared round or oval, well-defined, intense, hypervascular lesions in the arterial phase followed by the washout of contrast in the portal-delayed phases. All image reviews were performed using a picture archiving and communications system (PACS; Maroview, version 5.4, Infinitt) running on a workstation (XW6200, Hewlett-Packard) with monitors with a spatial resolution of 1600 x 1200.

Two additional radiologists with four and 20 years of clinical experience in interventional imaging and who did not participate in the diagnostic performance study further analyzed the formal CT reports and imaging features of all 297 hypervascular HCCs seen on the initial CT or CBCT-HA. The formal CT reports were retrospectively reviewed based on the detection of HCC. The two radiologists assessed the enhancement patterns of the hypervascular HCCs seen on contrast-enhanced, dynamic CT and categorized it into three types: Type I was defined as typical HCC showing arterial enhancement and portal or delayed washout; Type II was defined as atypical HCC showing arterial enhancement without portal or delayed washout; and Type III was defined as atypical HCC showing only portal or delayed washout without arterial enhancement.

### Statistical analysis

The diagnostic performance of each reviewer and the formal CT report were assessed using receiver operating characteristic (ROC) analysis. Sensitivity for the detection of HCC was determined by the number of lesions assigned a confidence level of 4 or 5 among the 297 HCCs. We also assessed the sensitivity and specificity of the radiologic diagnosis for HCC based on the AASLD and the Asian Pacific Association for the Study of the Liver (APASL) guidelines while applying our methods to confirm hypervascular HCC. According to the AASLD guidelines [1], nodules larger than 1 cm and with a typical enhancement pattern should be considered as HCC. Conversely, typical HCC can be diagnosed regardless of size if the characteristic imaging patterns are seen on dynamic CT according to the APASL guidelines [24].

The level of agreement between the two reviewers on the confidence scale regarding the possibility of HCC was measured using к statistics; therefore, weighted κ values <0 indicated no agreement; 0<κ≤0.2, slight agreement; 0.2<κ≤0.4, fair agreement; 0.4<κ≤0.6, moderate agreement; 0.6<κ≤0.8, substantial agreement; and 0.8<κ≤1, almost perfect agreement. Statistical analyses were performed using SPSS software (version 19.0; SPSS, Chicago, IL, USA) and MedCalc software (version 12.4.0.0; MedCalc Software, Mariakerke, Belgium).

## Results

### Enhancement patterns and the detection rate of hypervascular HCCs according to their size

**Fig 2:**
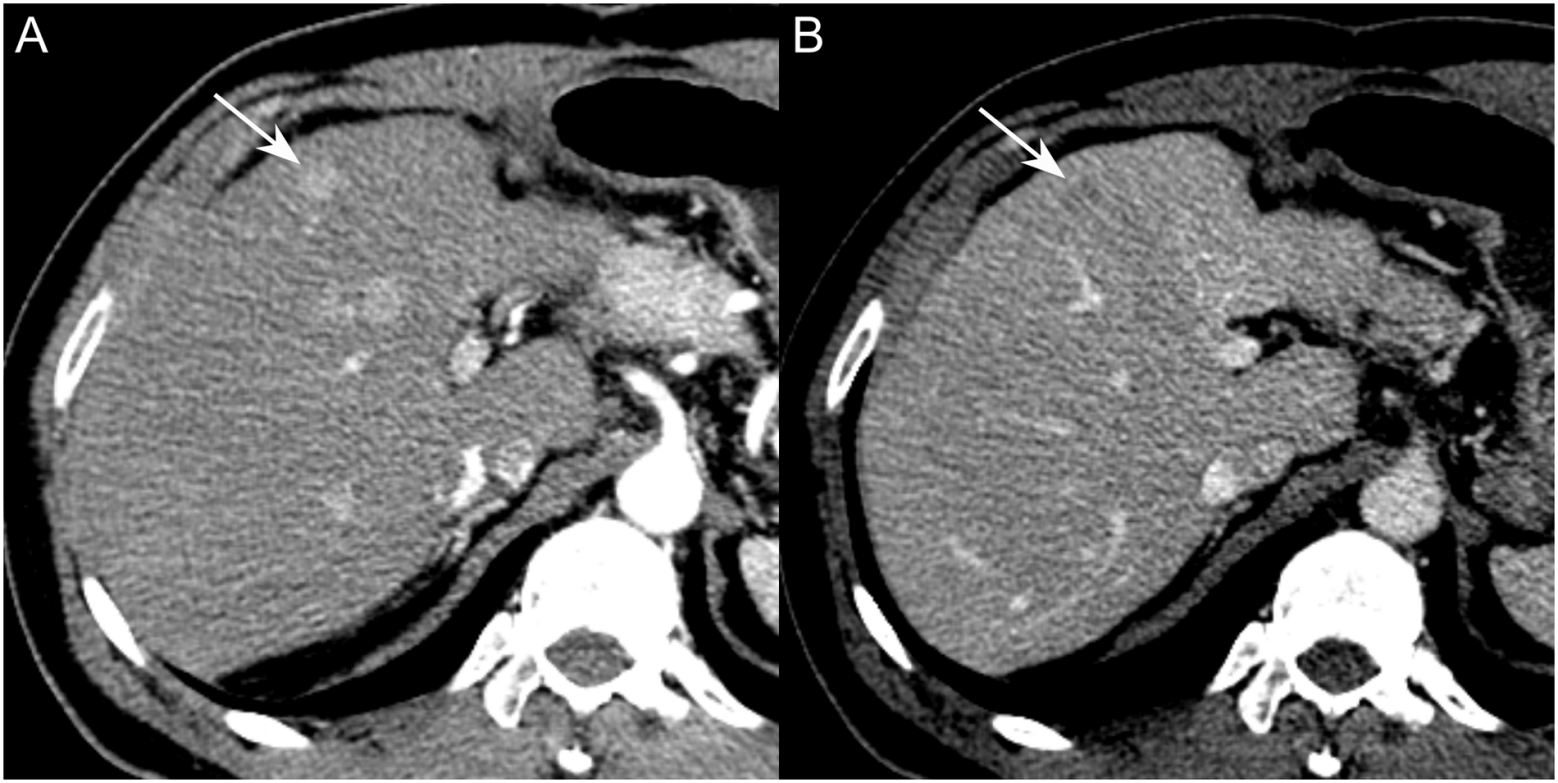
58-year-old man with type I HCC larger than 1 cm in diameter. **A-B**, On the contrast-enhanced CT in arterial (**A**) and portal venous (**B**) phases images, a 14 mm sized nodule in S4 of the liver showed arterial enhancement and washout (**type I**). This lesion was reported as HCC in the formal report, and both reviewers also assessed this lesion as HCC. After TACE, compact nodular uptake of Lipiodol was seen on the post-TACE and follow-up CT (not shown).

Among the 297 initially detected HCC lesions in 99 patients, 40 patients had a single lesion, 24 had two lesions, 11 had three lesions, seven had four lesions, 14 had five to 10 lesions, and three had more than 10 lesions detected on CBCT-HA. Of the 297 hypervascular HCCs, 142 (47.8%) were more than or equal to 1 cm in size and 155 (52.2%) were less than 1 cm in size. According to the enhancement patterns, 154 HCCs (51.9%) were categorized as type I, i.e., arterial enhancement and portal/delayed washout, (Fig 2), 53 HCCs (11.8%) as type II, i.e., arterial enhancement without portal/delayed washout, and 22 HCCs (7.4%) as type III, i.e., only portal/delayed washout without arterial enhancement. The remaining 68 HCCs (22.9%) were not visualized on the initial dynamic CT (all < 1 cm, mean diameter of 5.9 ± 2.4 mm). Table 1 summarizes the enhancement patterns and detection rates of HCC according to size.

**Table 1:** Enhancement patterns and detection rates of HCC according to size.

Enhancement Pattern Size	Lesions	Detection Rate		
		Formal Report	Reviewer 1	Reviewer 2
**Type I**	**154**	120 (77.9%)	115 (74.7%)	123 (79.9%)
**≥ 1 cm**	114	102 (89.5%)	98 (86%)	105 (92.1%)
**< 1 cm**	40	18 (45%)	17 (42.5%)	18 (45%)
**Type II**	**53**	21 (39.6%)	14 (26.4%)	17 (32.1%)
**≥ 1 cm**	16	10 (62.5%)	8 (50%)	9 (56.3%)
**< 1 cm**	37	11 (29.7%)	6 (16.2%)	8 (21.6%)
**Type III**	22	9 (40.9%)	7 (31.8%)	5 (22.7%)
**≥ 1 cm**	12	6 (50%)	3 (25%)	4 (33.3%)
**< 1 cm**	10	3 (30%)	4 (40%)	1 (10%)
**Non-visualized**	68			
**≥ 1 cm**	0	0	0	0
**< 1 cm**	68			
**Total**	297	149 (50.2%)	137 (46.1%)	145 (48.8%)
**≥ 1 cm**	142	118 (83.1%)	109 (76.8%)	118 (83.1%)
**< 1 cm**	155	32 (20.6%)	27 (17.4%)	27 (17.4%)

For 154 type I HCCs, the detection rates indicated in the formal CT report by reviewer 1 and reviewer 2 were 77.9% (120/154), 74.7% (115/154), and 79.9% (123/154). With regard to size, the detection rates of HCCs ≥ 1 cm were 83.1% (118/142), 76.8% (109/142), and 83.1% (118/142) in the formal report for reviewer 1 and reviewer 2. Alternatively, the detection rates of HCCs < 1 cm were 20.6% (32/155), 17.4% (27/155), and 17.4% (27/155) in the formal report for reviewer 1 and reviewer 2.

### Diagnostic performance of CT for hypervascular HCCs

**Fig 3:**
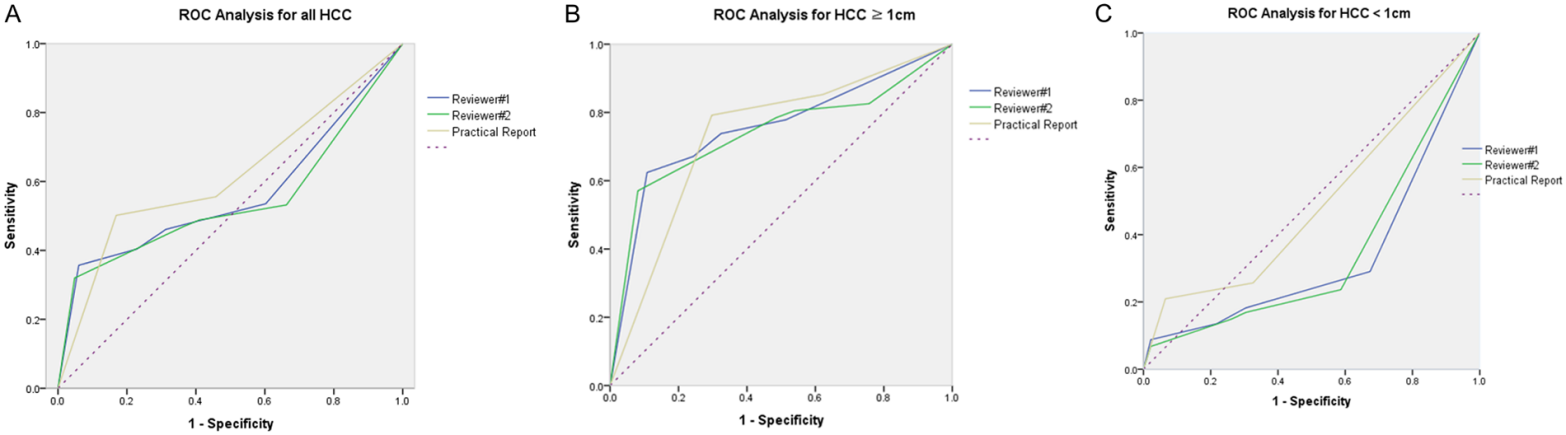
Diagnostic performance of CT for HCC. **A**. The area under the ROC curve for all HCC was 0.617, 0.561, and 0.542 in the formal CT report, reviewer 1, and reviewer 2. **B**. The area under the ROC curve for HCC >1cm was 0.735, 0.754, and 0.739 in the formal CT report, reviewer 1, and reviewer 2. **C**. The area under the ROC curve for HCC <1cm was 0.509, 0.666 and 0.654 in the formal CT report, reviewer 1, and reviewer 2.

Table 2 summarizes the diagnostic performance of CT for hypervascular HCC. The diagnostic performance using the area under the ROC curve (AUC) of HCCs ≥ 1 cm was 0.735, 0.754, and 0.739 for reviewer 1 and reviewer 2 in the formal CT report. Alternatively, the diagnostic performances of HCCs < 1 cm were 0.491, 0.334, and 0.346 in the formal CT report for reviewer 1 and reviewer 2 (Fig 3). The positive predictive values (PPVs) of HCCs ≥ 1 cm were 91.5%, 90.2%, and 85.7% in the formal report for reviewer 1 and reviewer 2. Alternatively, the PPVs of HCCs < 1 cm were 73.2%, 65.9%, and 64.1% in the formal report for reviewer 1 and reviewer 2. The interreader agreement between reviewers 1 and 2 was moderate, i.e., к = 0.58 for ≥ 1 cm, к = 0.42 for < 1cm, and к = 0.57 in total.

**Table 2:** Diagnostic performance of CT for HCC.

	AUC	Sensitivity (%)	Specificity (%)	PPV	NPV
**Formal Report**					
All HCC (n = 297)	0.617	50.2	83.13	91.4	
≥ 1 cm (n = 142)	0.735	79.2	70.3	91.5	*
≥ 2 cm (n = 46)	0.625	100.0	25.0	93.9	
1–2 cm (n = 96)	0.768	75.0	66.7	86.7	
< 1 cm (n = 155)	0.491	27.2	67.4	73.2	
**Reviewer 1**					
All HCC (n = 297)	0.561	46.1	68.7	84.0	26.3
≥ 1 cm (n = 142)	0.754	73.8	67.6	90.2	39.1
≥ 2 cm (n = 46)	0.723	95.7	50.0	95.7	50.0
1–2 cm (n = 96)	0.704	67.7	69.7	86.7	42.6
< 1 cm (n = 155)	0.334	18.2	69.6	65.9	20.9
**Reviewer 2**					
All HCC (n = 297)	0.542	48.8	59.0	81.0	24.4
≥ 1 cm (n = 142)	0.739	80.5	46.0	85.7	37.0
≥ 2 cm (n = 46)	0.500	100.0	0	92.0	-
1–2 cm (n = 96)	0.648	75.0	51.5	81.8	41.5
< 1 cm (n = 155)	0.346	16.9	69.6	64.1	20.7

Note. AUC means values of an area under the response operating characteristic curve, PPV means positive predictive value (%), and NPV means negative predictive value (%)

*The NPV in the formal report could not be evaluated because the lesions considered as benign were not always specified by the formal report

**Fig 4:**
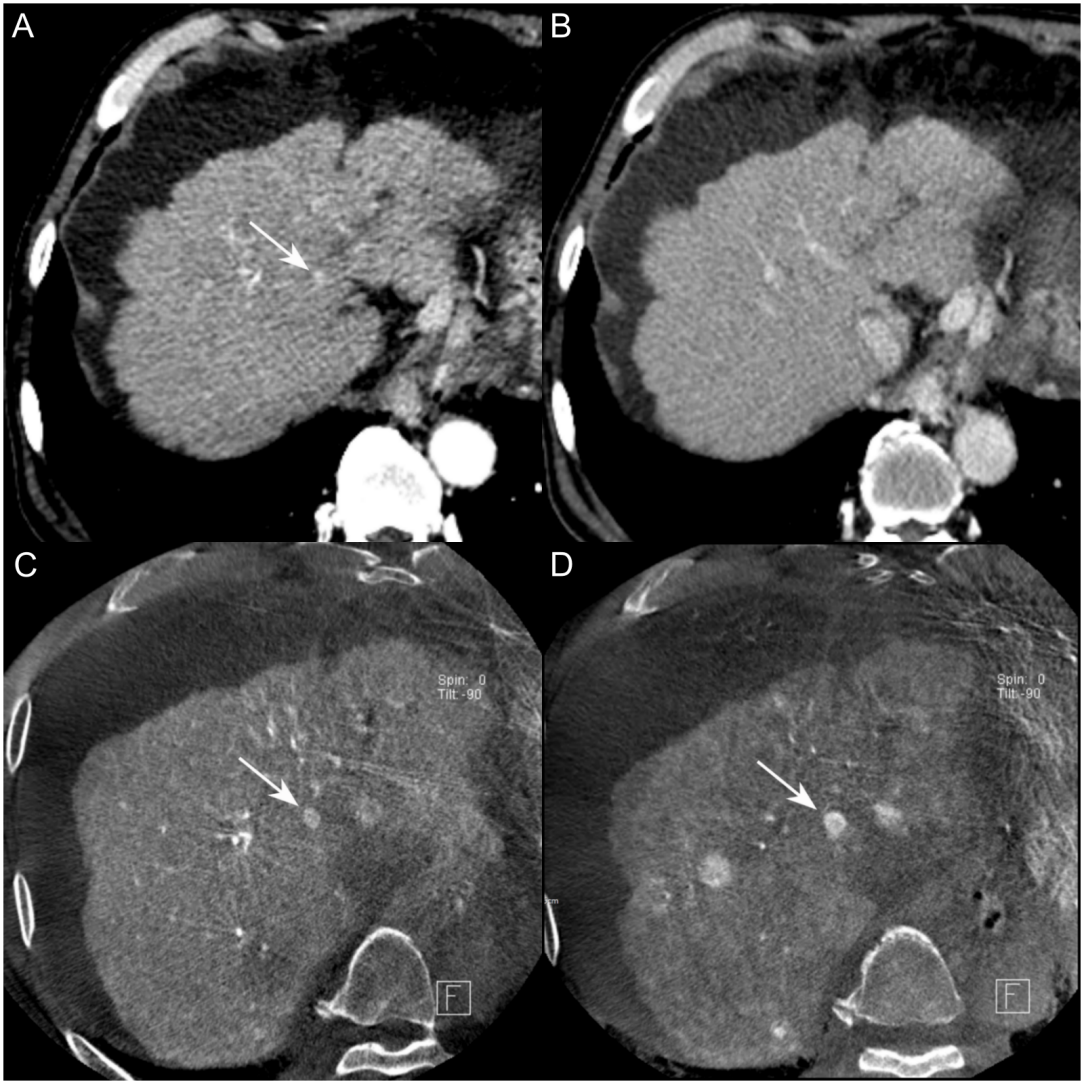
76-year-old man with type II HCC smaller than 1 cm in diameter. **A-B**, On the contrast-enhanced CT in arterial (**A**) and delayed (**B**) phases images, a 7 mm sized nodule in S1 of the liver showed arterial enhancement without washout (type II, arrow). This lesion was not mentioned on formal CT report, and was not detected by both reviewers, neither. **C-D**, CBCT-HA images on initial TACE session (**C**) and 6 months later (**D**). A small hypervascular nodule showed interval growth (11 mm, arrow) on follow up CBCT-HA. This nodule showed interval growth with typical enhancement pattern of HCC on the follow-up CT (not shown).

The numbers of false-positive nodules in the formal report for reviewer 1 and reviewer 2 were 14, 26, and 34. The numbers of false-negative nodules in the formal report and for reviewers 1 and 2 were 147, 160, and 152. Among the false negative results, 83.7%, 93.8%, and 97.4% in the formal report for reviewers 1 and 2 were smaller than 1 cm. With the exception of nonvisualized nodules (n = 68) among the false-negative nodules, 32 (40.5%), 39 (42.4%), and 36 (42.9%) showed the type II enhancement pattern, i.e., arterial enhancement without portal/delayed washout (Fig 4), and 10 (12.7%), 13 (14.1%), and 13 (15.5%) lesions showed the type III enhancement pattern, i.e., only portal/delayed washout without arterial enhancement, in the formal report and for reviewers 1 and 2.

When applying our results to current AASLD guidelines, the sensitivity and specificity of CT for diagnosing hypervascular HCC ≥ 1 cm were 80.3% (114/142) and 89.2% (33/37), respectively. For hypervascular HCCs of all sizes, the sensitivity was 51.9% (154/297) and the specificity was 89.2% (74/83) based on the APASL guidelines.

## Discussion

Subcentimeter-sized HCCs accounted for 52.2% of the total of 297 hypervascular HCCs according to our method for diagnosing hypervascular HCC using CBCT-HA and CT during a two-year follow-up period. A relatively high percentage of subcentimeter-sized HCCs indicates that imaging diagnosis using contrast-enhanced, dynamic CT may frequently miss small, < 1 cm, hypervascular HCCs.

In our study, the detection rates for lesions between 1 cm and 2 cm were 75%, 68%, and 75% (formal report for reviewer 1 and reviewer 2) and were slightly lower than those for lesions ≥ 1 cm (79.2%, 73.8%, and 80.5%); however, the detection rates were significantly lower in lesions < 1 cm (27.2%, 18.2%, and 16.9%). Thus, the low sensitivity of detecting small HCC was mainly caused by lesions < 1 cm rather than < 2 cm. In addition, all 68 HCCs invisible on CT were less than 1 cm in diameter. In conclusion, improved CT resolution has increased the detection of small nodules, but CBCT-HA demonstrates that it still missed a large number of nodules less than 1 cm in diameter.

Our study demonstrated the role of CBCT-HA in depicting small, hypervascular HCCs that may not be evident on CT. Using CBCT-HA during chemoembolization, 74 HCCs (24.9%) were additionally detected (Group 2), and another 74 HCCs (24.9%) were further confirmed after interval growth (Group 3). Owing to the high spatial resolution and more selective intra-arterial bolus injection of contrast media, CBCT-HA showed higher detectability of small hypervascular HCC. Iwazawa et al. [25] showed that the accuracy of CBCT-HA was significantly greater than that of MDCT for all HCCs, i.e., MDCT, 0.785; CBCT-HA-HA, 0.869; and p = 0.003, and for small HCCs < 1 cm, i.e., MDCT, 0.618; CBCT-HA-HA, 0.830; and p < 0.001). Our study also demonstrated the higher sensitivity for detecting small, hypervascular HCCs on CBCT-HA during TACE as 68 HCCs were only seen on CBCT-HA; therefore, additional detection and treatment of CT-negative HCC by CBCT-HA at the TACE session may reduce recurrence and increase patient survival.

Our study shows that of the 142 hypervascular HCCs 1 cm or larger in size, 114 met the AASLD guidelines, i.e., sensitivity = 80.3% and specificity = 89.2%; however, for hypervascular HCCs of all sizes based on the APASL guidelines, sensitivity was decreased to 51.9% (154/297). Additionally, only 154 HCCs (51.9%) showed the typical enhancement patterns of HCC including arterial enhancement and washout on the portal or delayed phase. Because of atypical enhancement patterns, the detection rates of HCCs ≥ 1 cm were 83.1% (118/142), 76.8% (109/142), and 83.1% (118/142) for both reviewer 1 and reviewer 2 in the formal report. The detection rates became much lower, especially in the subcentimeter-sized hypervascular HCCs, and were 20.6% (32/155), 17.4% (27/155), and 17.4% (27/155) in the formal report for reviewer 1 and reviewer 2. Our results showing particularly low sensitivity for small, hypervascular HCCs are comparable to those of other published studies [26–34]. In a previous study by Baek et al. [27], sensitivities in the detection of HCCs < 1 cm using MDCT dropped from 89.8% and 91.5% to 14.3% and 21.4% when compared to those of HCCs ≥ 1 cm. In addition, 27 out of 34 (79.4%) false-negative results regarding the diagnosis of HCC using MDCT images were smaller than 1 cm in their study. Kim et al. [34] also reported the lower sensitivities for HCCs < 1 cm when using MDCT compared to HCCs ≥ 1 cm according to the three observers (30–50% vs 93.2–97.2%).

In our study, typical enhancement patterns accounted for 80.3% (114/142) in hypervascular HCCs ≥ 1 cm and 25.8% (40/155) in hypervascular HCCs < 1 cm. Out of HCC lesions < 1 cm, 43.9% were not discriminable on CT. Similarly, in the previous report by Luca et al. [29], the rate of typical findings increased according to the increasing size of the HCCs where the rate of atypical nodules and undetectable HCC increased according to the decreasing size of the HCCs. They showed that the overall sensitivity of CT was 89% for a total of 131 HCCs detected on liver explants, although using typical enhancement patterns resulted in a sensitivity of 43%.

Despite the recent advent of multidetector CT (MDCT) and many technical advances, the diagnostic accuracy for identifying small HCCs is still unsatisfactory because of the difficulty in detecting and characterizing small nodules with faint or atypical enhancement. They must also be differentiated from benign lesions, such as regenerative nodules, small hemangiomas or arterioportal shunts, especially when the washout is vague on dynamic CT. Shimizu et al.[35] showed that among the small (≤ 2 cm) round or oval lesions 52% disappeared or decreased in size and were considered to be pseudolesions, and only 28% were classified as HCC. Accordingly, false-positive results, as well as detectability, would be problematic in diagnosing small HCCs on CT.

There are some limitations to our study. First, there may have been a patient selection bias due to the retrospective study design. Second, our method for diagnosing hypervascular HCC was not based on the pathologic diagnosis of HCC; however, characteristic imaging features, compact iodized oil uptake on the follow-up CT, and the interval growth with typical imaging features on follow-up CT or MRI allowed us to establish the reference standards for hypervascular HCC. In particular, a two-year follow-up period might be sufficient for detecting small, slowly growing HCCs that are not visualized on dynamic CT or MRI but are seen on CBCT-HA as small, hyperenhancing nodules.

In conclusion, according to our method to confirm hypervascular HCC, multiple subcentimeter-sized HCCs were frequently missed or not evident on CT. CT may be helpful for diagnosing HCCs ≥ 1 cm with typical enhancement patterns but has limitations for diagnosing HCCs <1 cm or with atypical enhancement patterns.
